# Supplementary material for: Predicting chromosomal compartments directly from the nucleotide sequence with DNA-DDA
Source: Brief Bioinform. 2023 Jun 1;24(4):bbad198. doi: 10.1093/bib/bbad198 (PMC10359093; doi:10.1093/bib/bbad198)
Supplement: Lainscsek_BriefBioinform_SupplementaryMaterial_Apr2023_bbad198 [file lainscsek_briefbioinform_supplementarymaterial_apr2023_bbad198.zip › Lainscsek_BriefBioinform_SupplementaryMaterial_Apr2023.pdf]

Predicting chromosomal compartments directly from the nucleotide sequence with DNA-DDA

Supplementary Material

Xenia Lainscsek and Leila Taher

Institute of Biomedical Informatics, Graz University of Technology

Figures

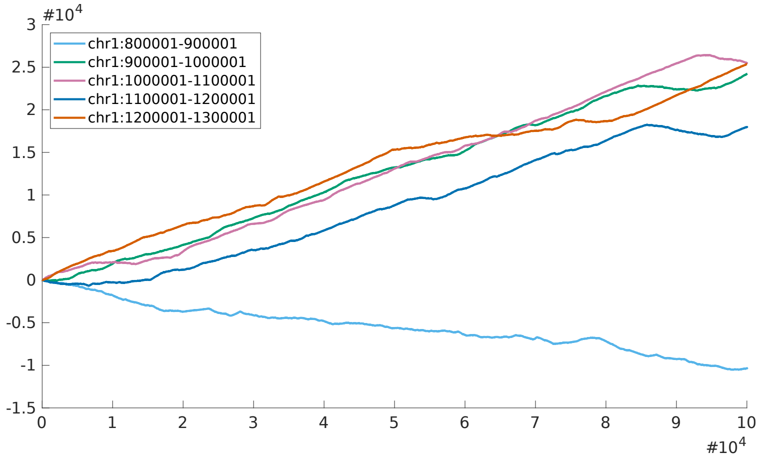

Figure S1: Random walk representation for first five bins of chr1

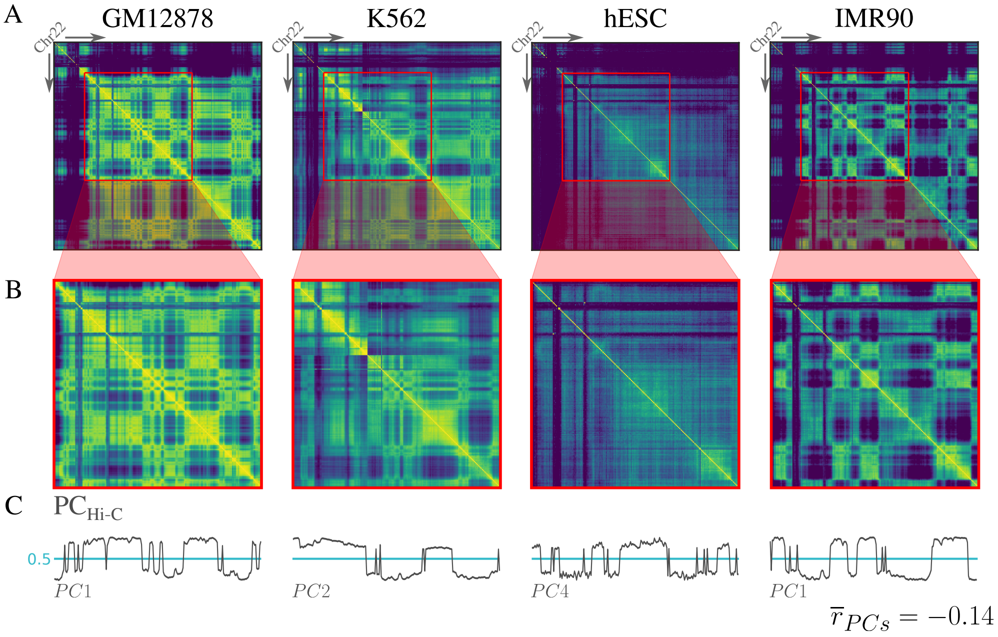

Figure S2: **Data used for supervised structure selection** A) The reference sequence of human chromosome 22 has 50,818,467 bps and can be partition into 509 100kbp-long bins. B) The 200 consecutive bins (chr22:16200000:36200001) with the C) highest variation (lowest Pearson's $\overline{r}_{low} = -0.14$) between cell types were selected to be used for structure selection. The PC number chosen to represent chromosomal compartments is based on their correlation with the H3K4me profile in the respective cell line. This can change depending on which region is being considered. Column two in the table indicates $PC_{chr22:1-50818468} \rightarrow PC_{chr22:16200000:36200001}$: K562 $PC_1 \rightarrow PC_2$; hESC $PC_1 \rightarrow PC_4$

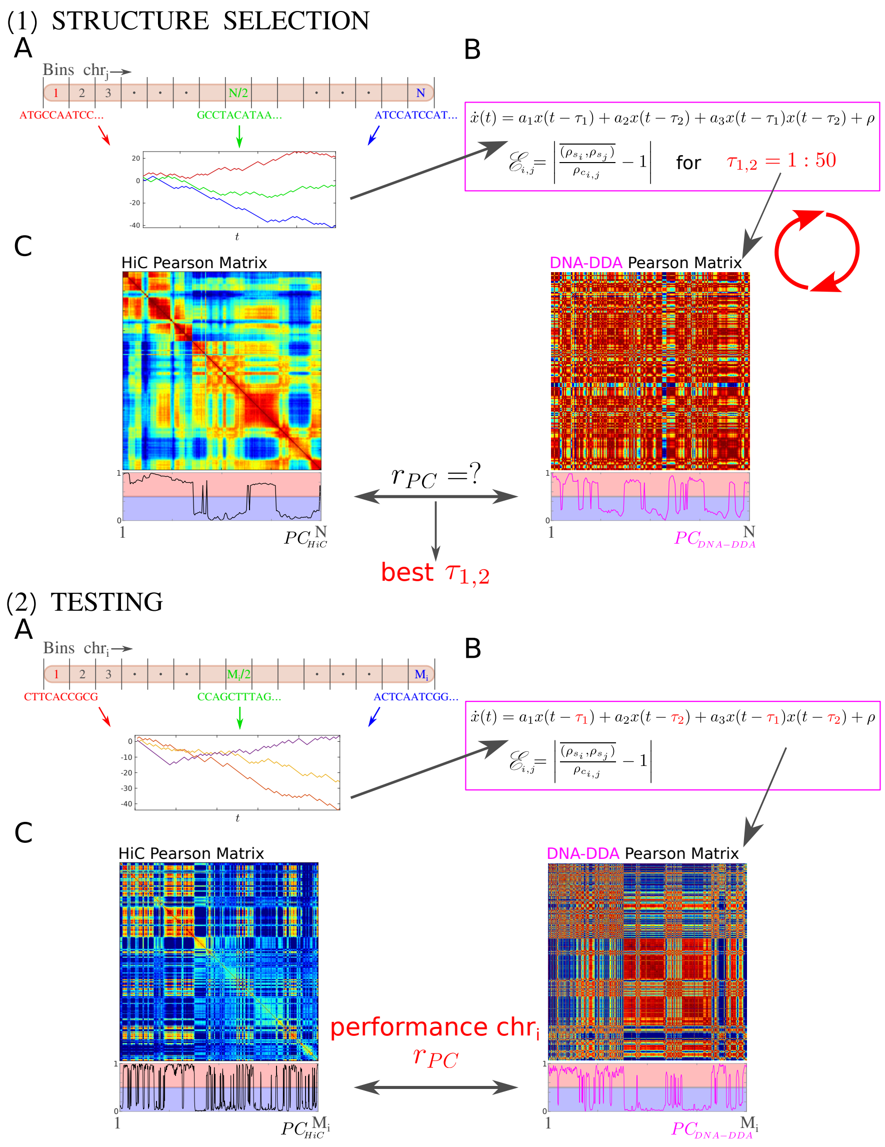

Figure S3: **Workflow for determining DNA-DDA parameters τ_1 and τ_2 and subsequent testing for an arbitrary cell line. 1) Structure Selection** 1A) Numerical encoding of the sequence as a 1D DNA walk $x(t)$ using structure selection data on chromosome j: $chr_j : 1 - N$ 1B) $x(t)$ is inputted into the DNA-DDA model; the ST and CT errors ρ_{ST}, ρ_{CT} are computed and combined to DE-DDA $\mathscr{E}$ (see Figure 1) for all delay pair combinations $\tau_1, \tau_2 \in [1 : 50]$ 1C) the best performing delay pair, defined as that which gives the highest Pearson correlation coefficient r_{PC} between $\text{PC}_{\text{Hi-C}}$ and $\text{PC}_{\text{DNA-DDA}}$, is fixed. **2) Testing** 2A) Numerical encoding of the sequence as a 1D DNA walk $x(t)$ for an arbitrary hold-out testing chromosome i: $chr_i : 1 - M$ 2B) $x(t)$ is inputted into the DNA-DDA model; the ST and CT errors ρ_{ST}, ρ_{CT} are computed and combined to DE-DDA $\mathscr{E}$ for best performing delay pair τ_1, τ_2 determined in step 1 2C) the final performance for DNA-DDA on chromosome i is given by r_{PC} between $\text{PC}_{\text{Hi-C}}$ and $\text{PC}_{\text{DNA-DDA}}$.

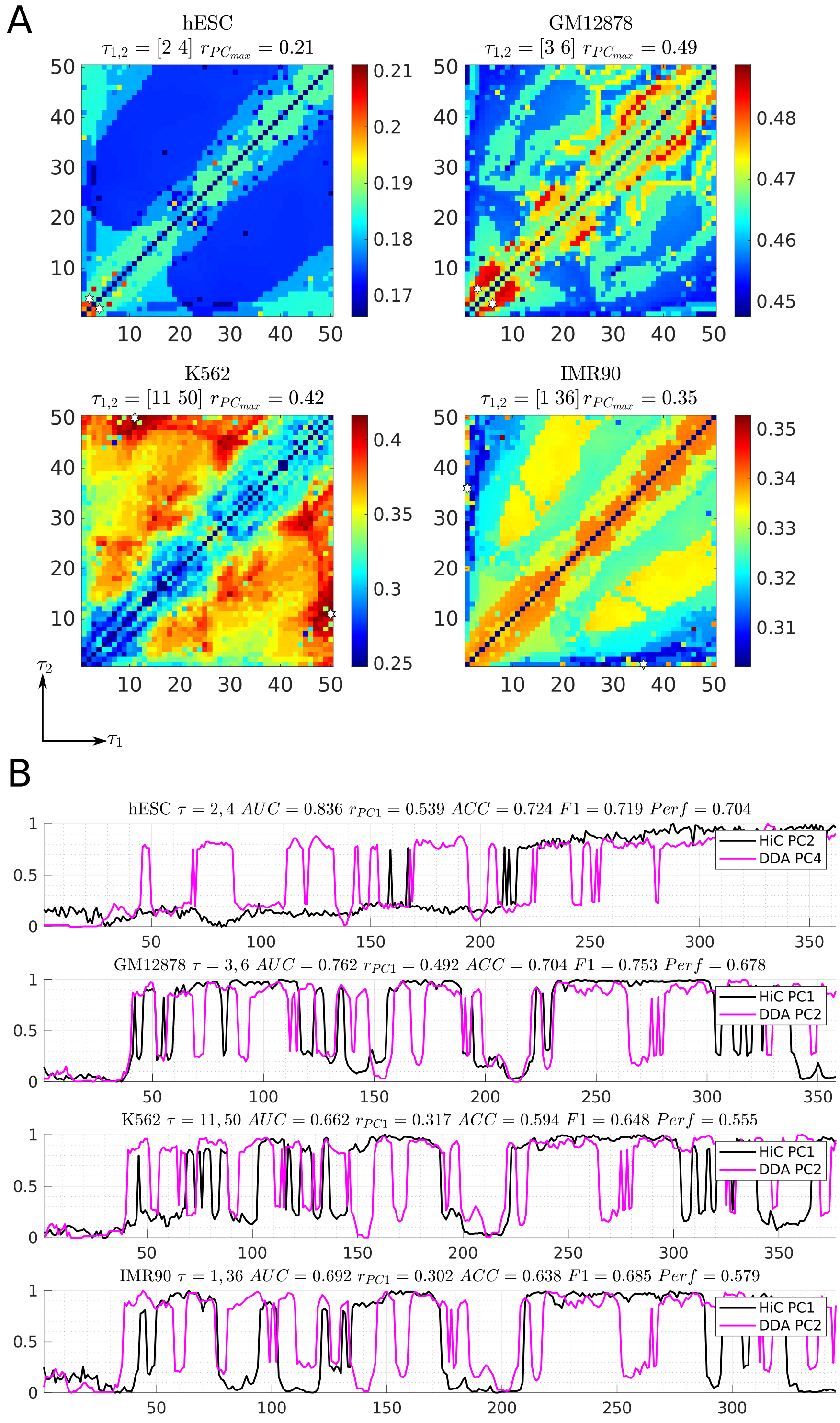

Figure S4: **Supervised structure selection on chr22 reveals regions of enriched dynamics across delay pairs and cell types.** A) Heatmaps depicting performance (Pearson correlation between $PC_{\text{DNA-DDA}}$ and $PC_{\text{Hi-C}}$) for each delay pair τ_1, τ_2 between 1 and 50 on genomic region chr22:16200000:36200001. The best performing delay pairs are indicated by a white hexagram. B) Performance of best performing delay pairs over entire chromosome 22.

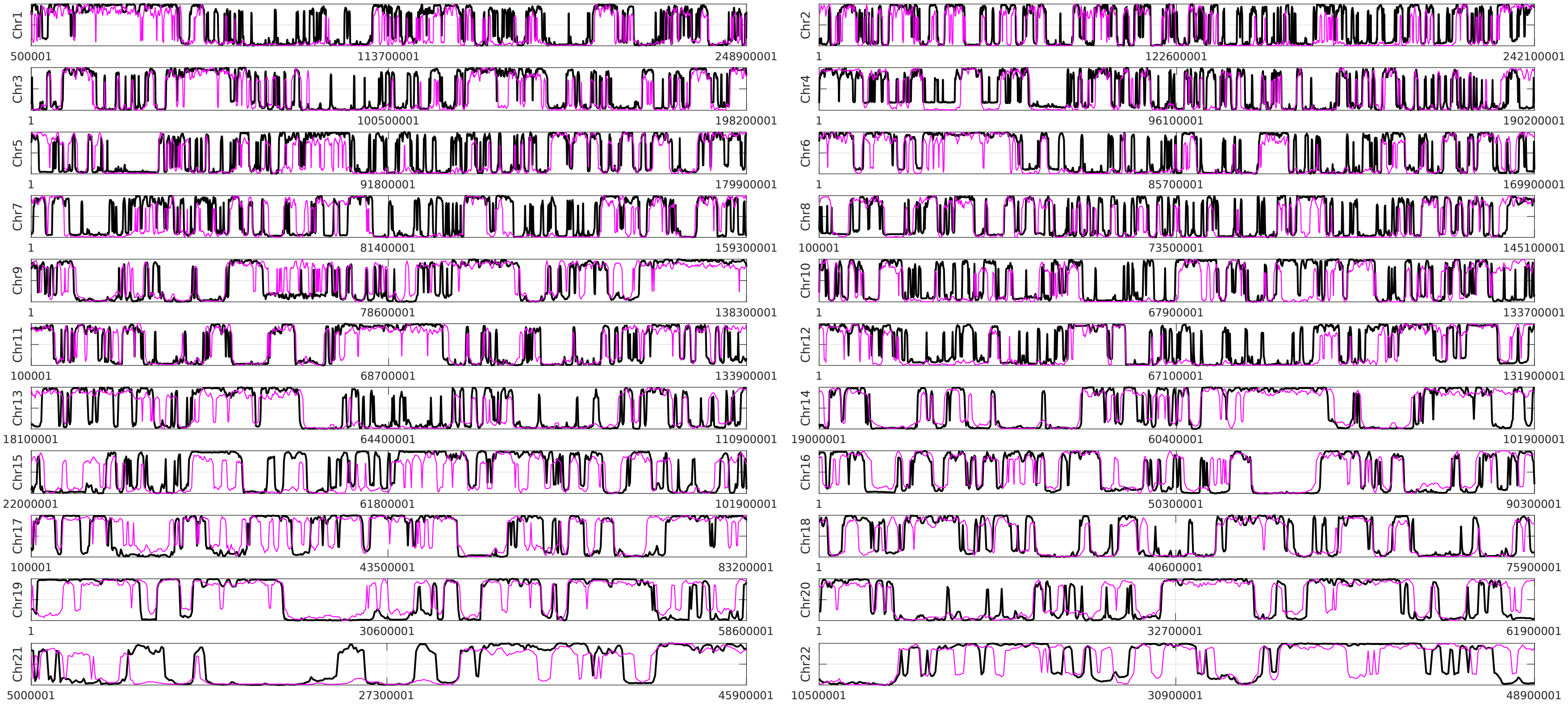

Figure S5: **$\mathbf{PC_{DNA\text{-}DDA}}$ (magenta) and $\mathbf{PC_{Hi\text{-}C}}$ (black) for GM12878 over each chromosome. normalized to 0 and 1.** Values above 0.5 correspond to the A compartment; values below 0.5 correspond to the B compartment.

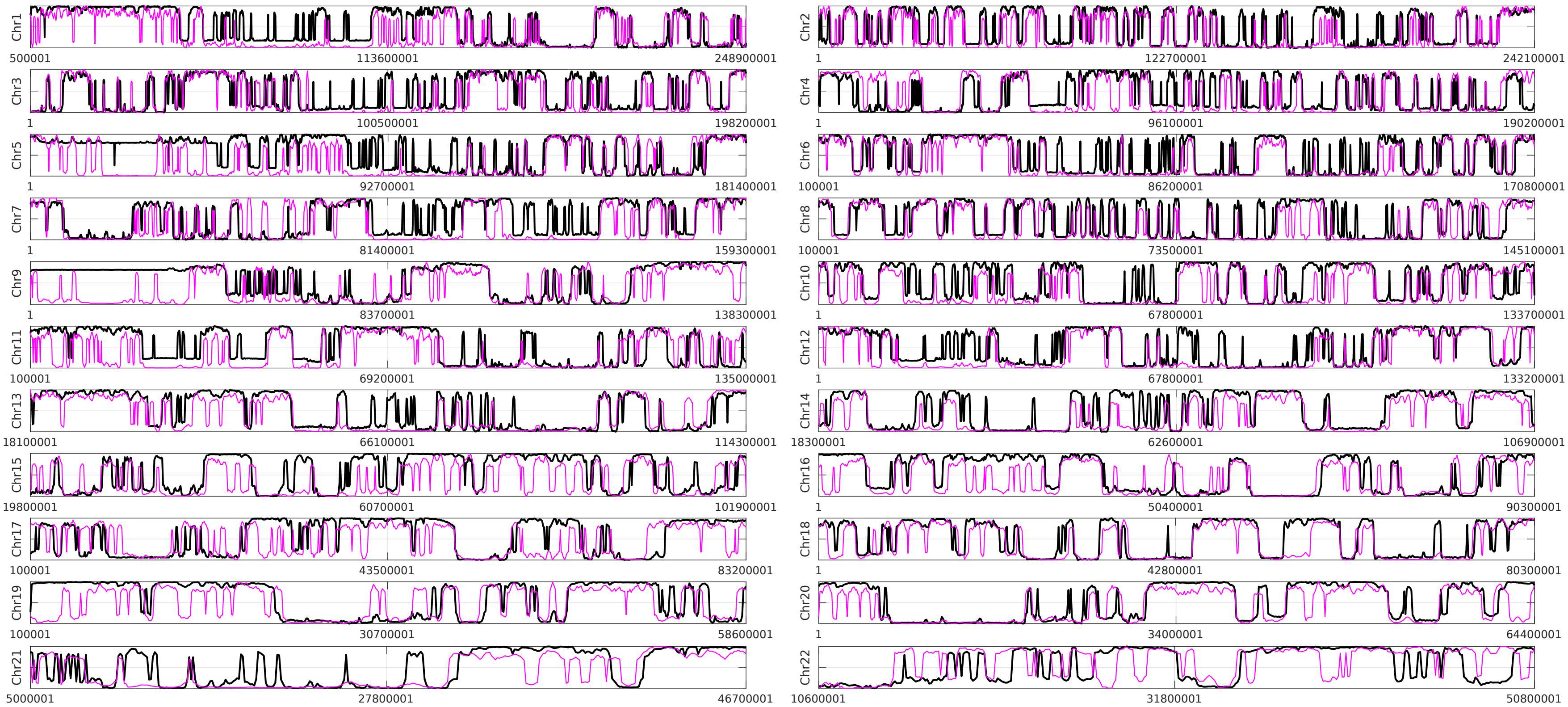

Figure S6: **$PC_{\text{DNA-DDA}}$ (magenta) and $PC_{\text{Hi-C}}$ (black) for K562 over each chromosome. normalized to 0 and 1.** Values above 0.5 correspond to the A compartment; values below 0.5 correspond to the B compartment.

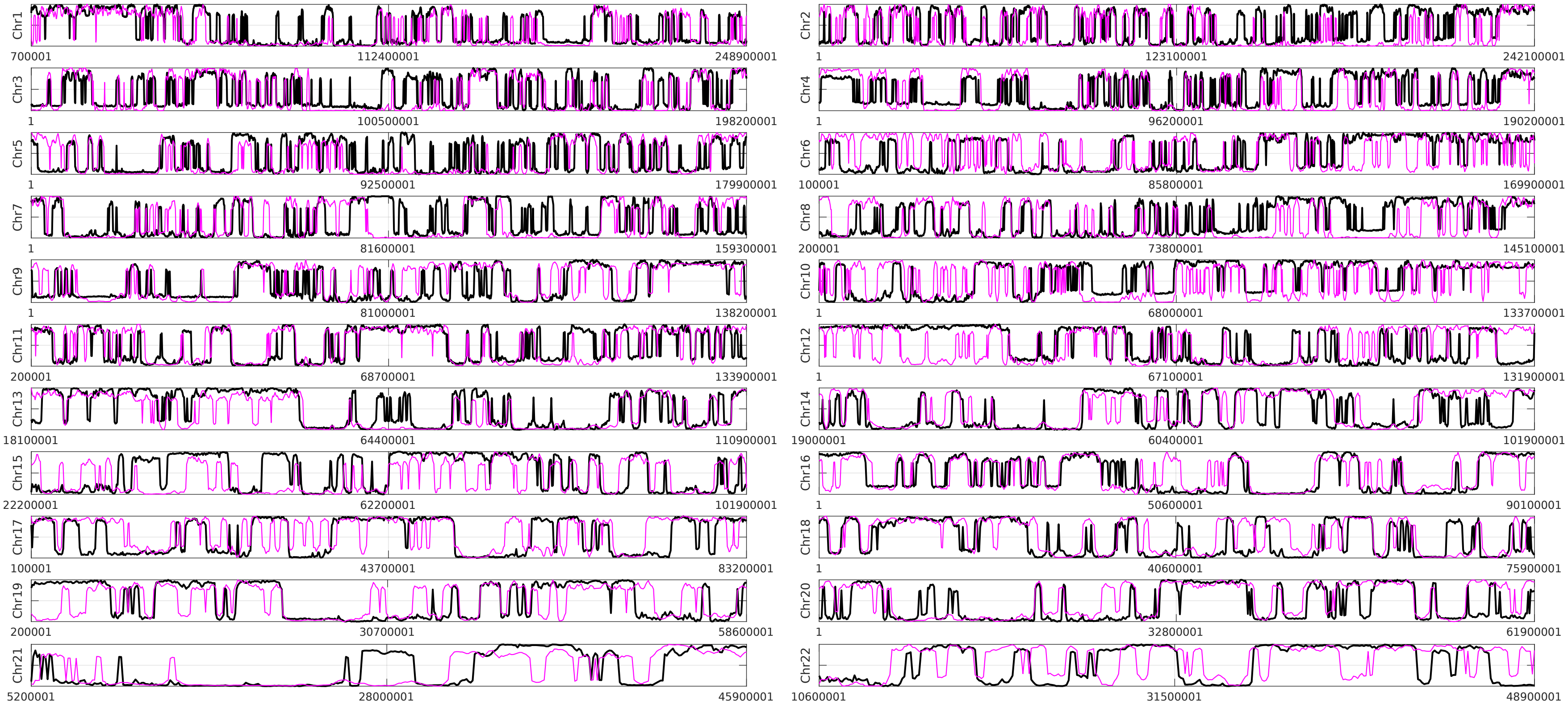

Figure S7: **$PC_{DNA\text{-}DDA}$ (magenta) and $PC_{Hi\text{-}C}$ (black) for IMR90 over each chromosome normalized to 0 and 1.** Values above 0.5 correspond to the A compartment; values below 0.5 correspond to the B compartment.

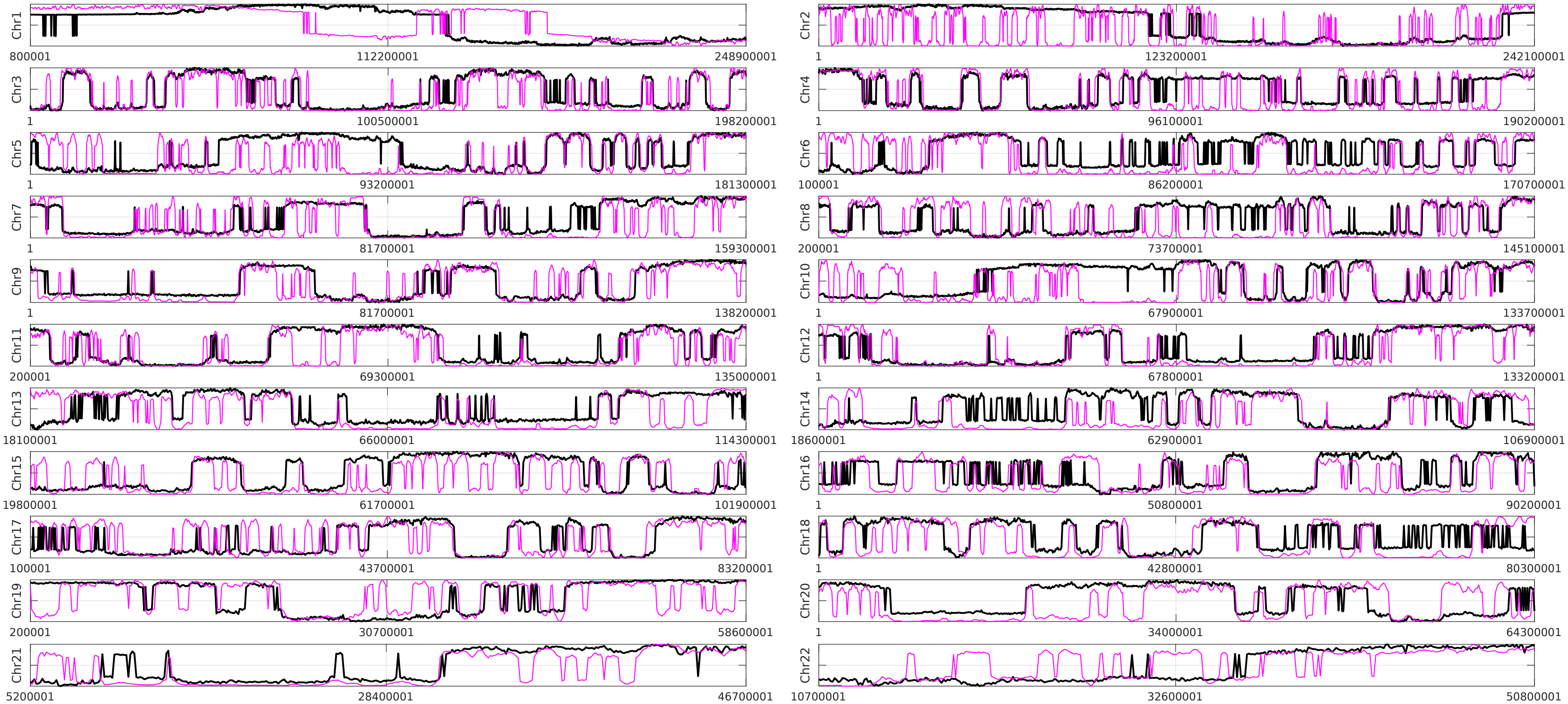

Figure S8: **$PC_{\text{DNA-DDA}}$ (magenta) and $PC_{\text{Hi-C}}$ (black) for hESC over each chromosome normalized to 0 and 1.** Values above 0.5 correspond to the A compartment; values below 0.5 correspond to the B compartment.

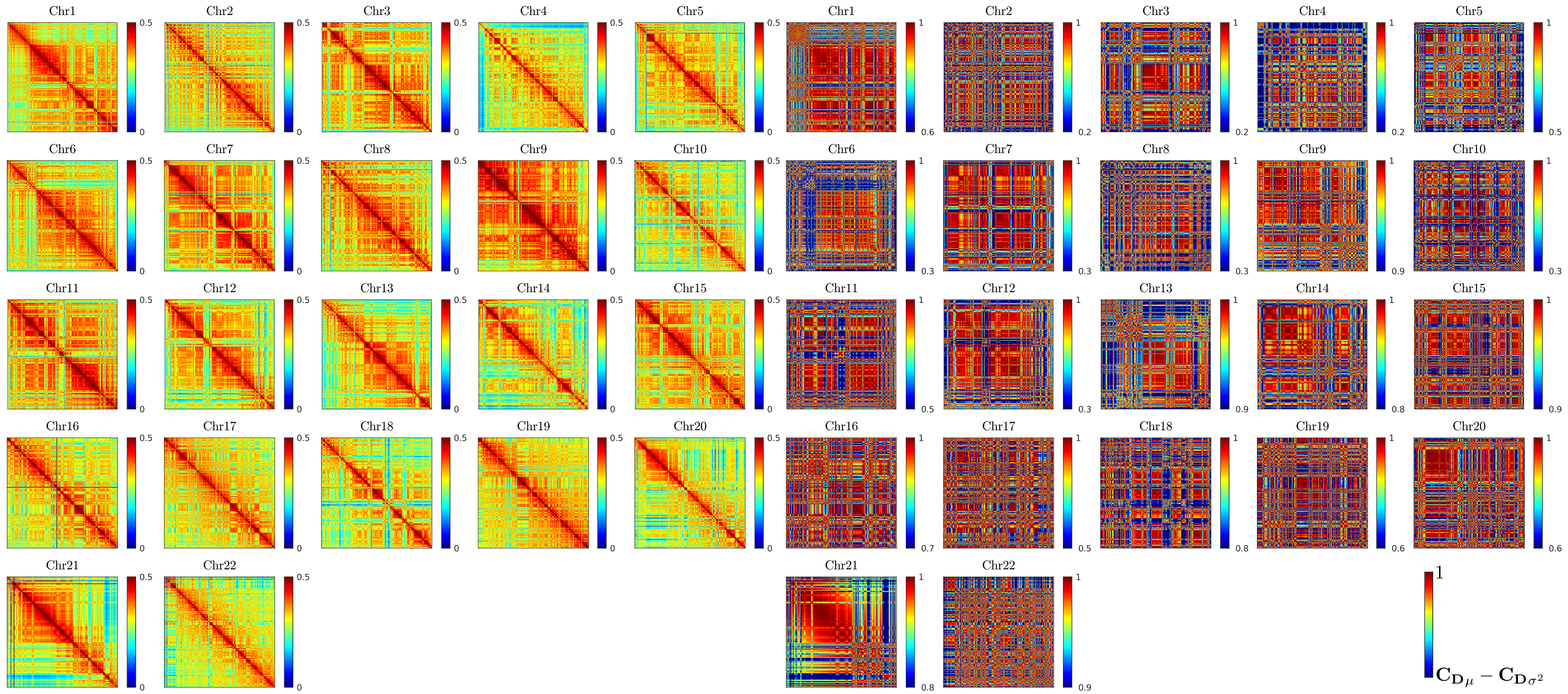

Figure S9: **(left) DNA-DDA contact maps D for GM12878.** Color scale goes from 0 to (right) DNA-DDA pearson contact maps $\mathbf{C}_\mathbf{D}$ for GM12878. Color scale goes from mean-variance $(\mathbf{C}_{\mathbf{D}\mu} - \mathbf{C}_{\mathbf{D}\sigma^2})$ to max

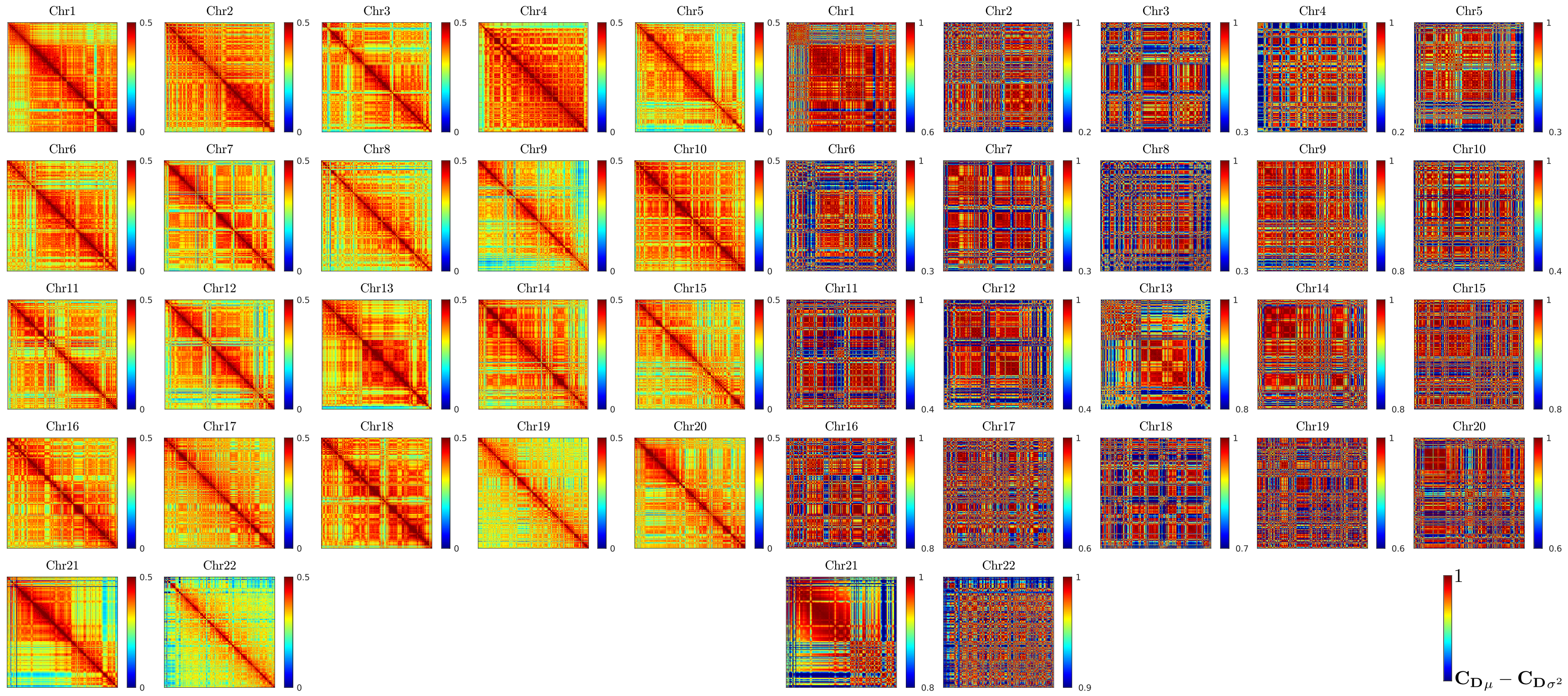

Figure S10: **(left) DNA-DDA contact maps D for K562.** Color scale goes from 0 to (right) DNA-DDA pearson contact maps $\mathbf{C_D}$ for K562. Color scale goes from mean-variance ($\mathbf{C_{D_\mu}} - \mathbf{C_{D_{\sigma^2}}}$) to max

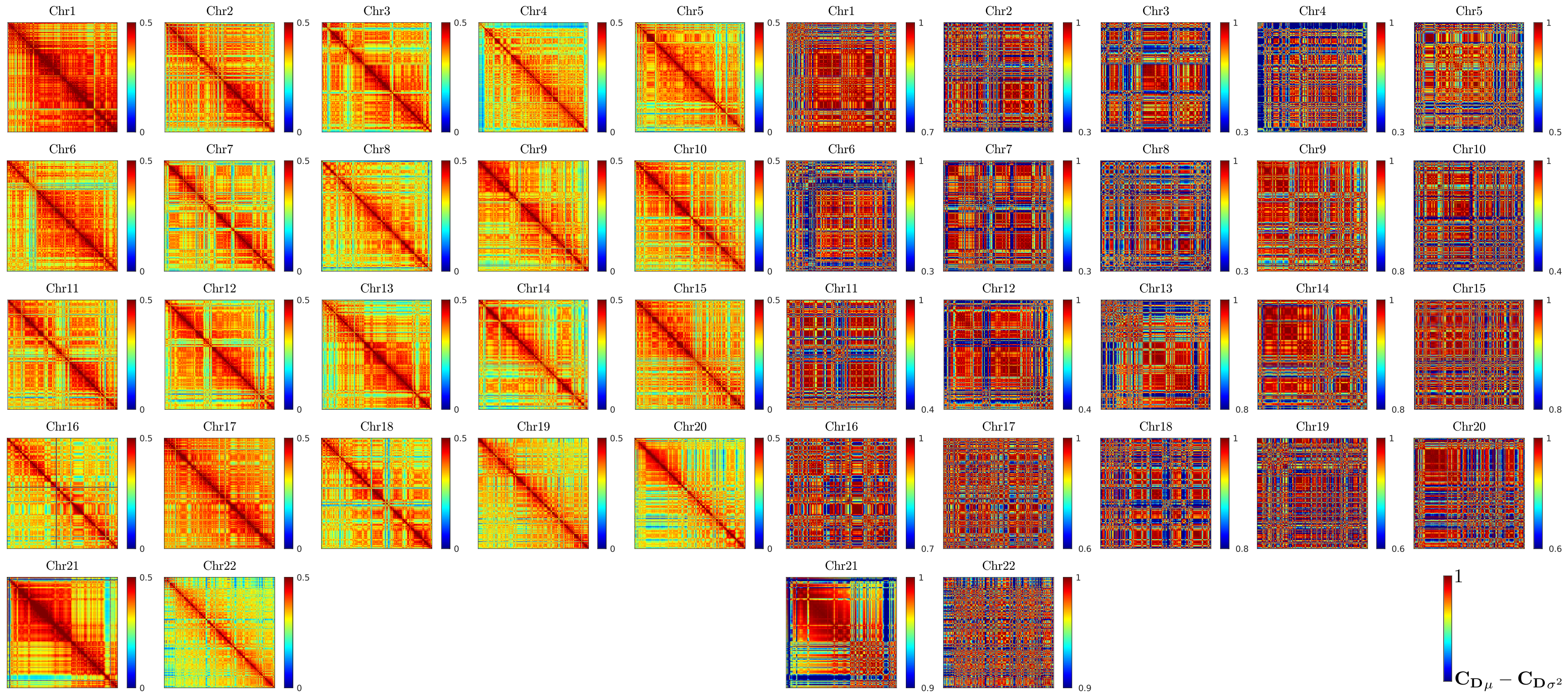

Figure S11: **(left) DNA-DDA contact maps D for IMR90.** Color scale goes from 0 to (right) DNA-DDA pearson contact maps $\mathbf{C_D}$ for IMR90. Color scale goes from mean-variance ($\mathbf{C_{D_\mu}} - \mathbf{C_{D_{\sigma^2}}}$) to max

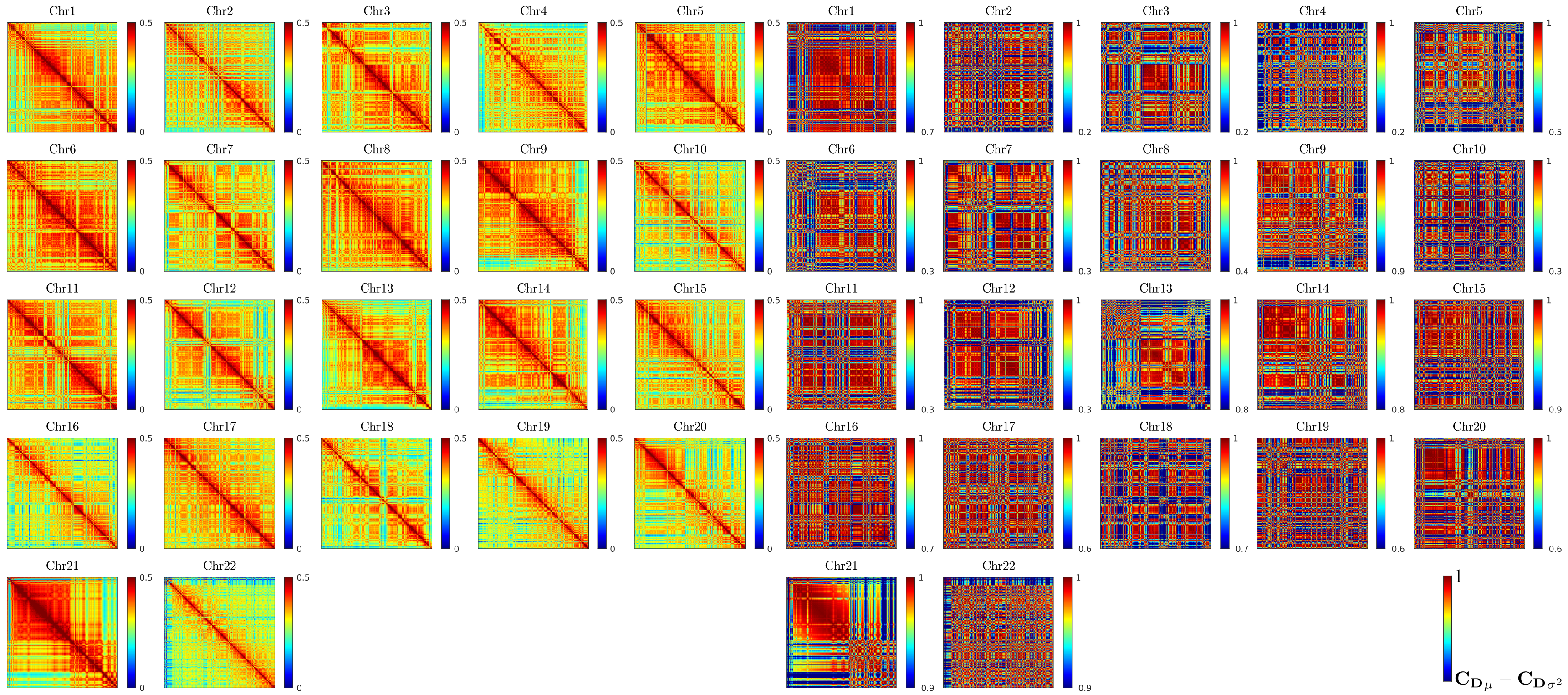

Figure S12: **(left) DNA-DDA contact maps D for hESC.** Color scale goes from 0 to (right) DNA-DDA pearson contact maps $\mathbf{C}_{\mathbf{D}}$ for hESC. Color scale goes from mean-variance ($\mathbf{C}_{\mathbf{D}\mu} - \mathbf{C}_{\mathbf{D}\sigma^2}$) to max

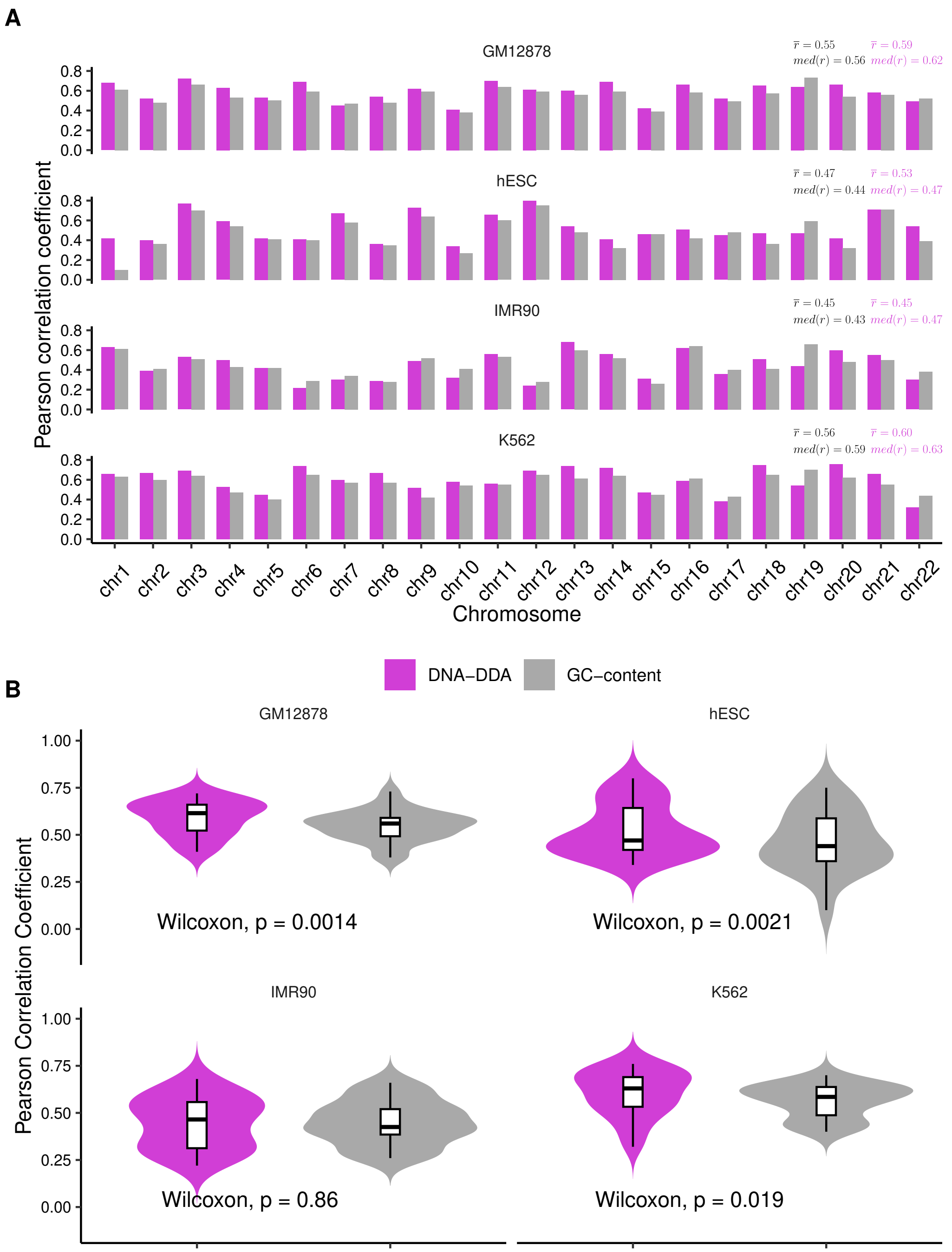

Figure S13: **DNA-DDA predictions are not only driven by the GC-content.** Pearson correlation coefficients between $PC_{\text{DNA-DDA}}$ and $PC_{\text{Hi-C}}$ compared to Pearson correlation coefficients between average GC-content and $PC_{\text{Hi-C}}$. Average GC-content was computed for the same consecutive non-overlapping 100kb-long genomic bins used for predicting compartments with DNA-DDA. A) Per chromosome. B) Per cell line. The P-value corresponds to the Wilcoxon signed-rank test. DNA-DDA outperforms the naïve GC-content-based compartment prediction.

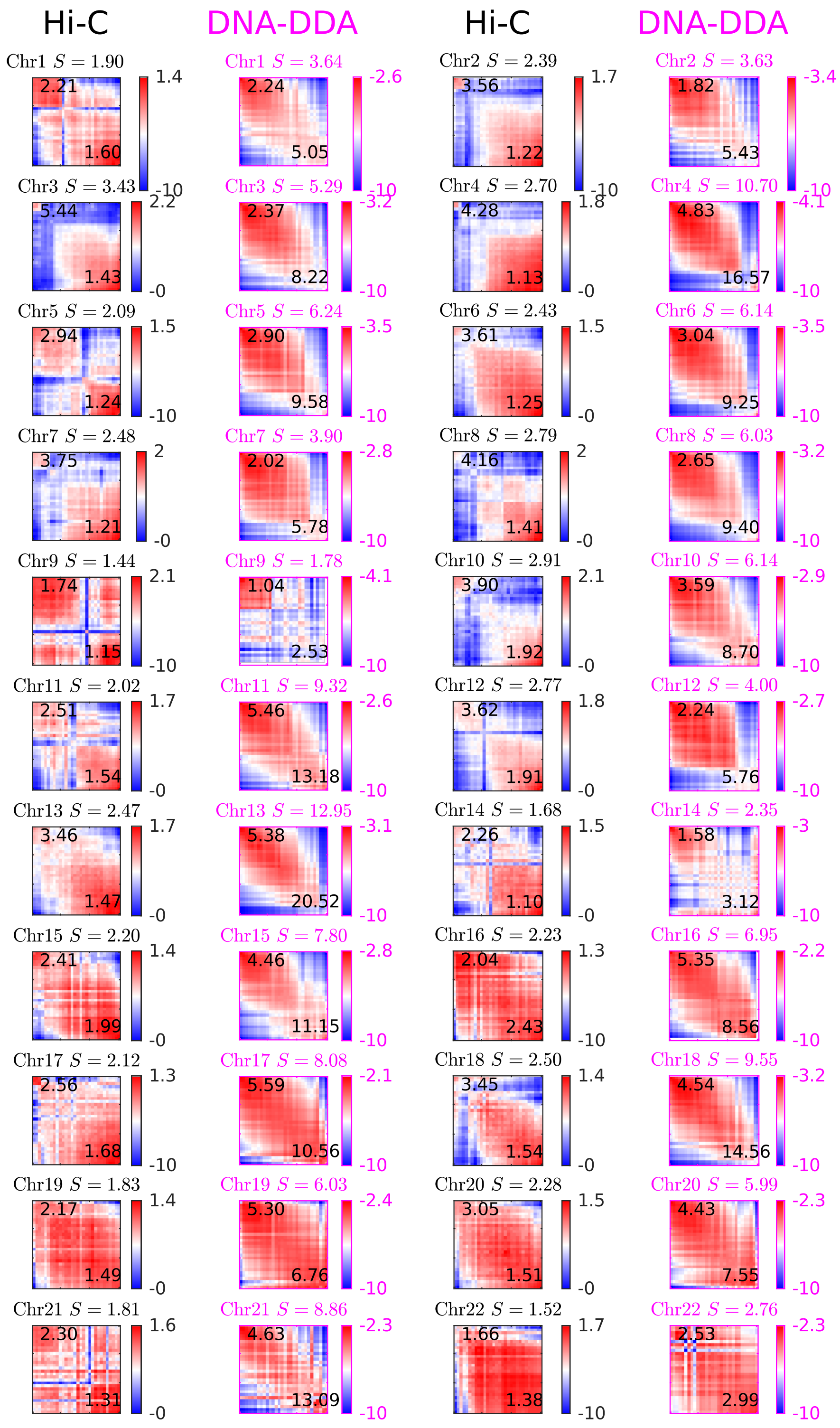

Figure S14: **Saddle plot analysis in K562.** Saddle plots derived from observed/expected Hi-C (black) and DNA-DDA (magenta) matrices. Colors indicate log2 of interaction values given by HiC_{OE} and $\mathbf{D}$ respectively. Compartment strengths AA, BB, AB and BA are shown in each corner and correspond to the highest (AA, BB) and lowest (AB, BA) 25% of the PC values. The overall saddle strength S is then calculated as $\frac{AA+BB}{AB+BA}$.

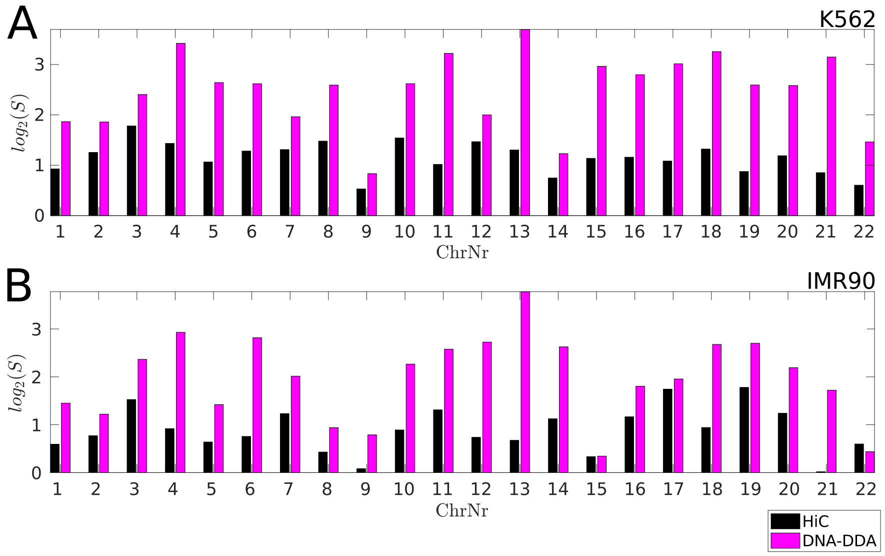

Figure S15: **DNA-DDA predicts overall stronger saddle strengths S.** Barplots of log2 saddle strengths across all chromosomes in A) K562 and B) IMR90.

Tables

Table S1: `--filterThreshold` parameter for normalizing contact matrices

Chr	GM12878		K562		IMR90		hESC	
	Z_{min}	Z_{max}	Z_{min}	Z_{max}	Z_{min}	Z_{max}	Z_{min}	Z_{max}
1	-2.0	2.4	-1.5	3.0	-0.7	2.0	-0.6	2.4
2	-2.0	2.6	-1.6	3.0	-0.8	2.0	-0.7	2.2
3	-2.0	2.6	-1.5	4.0	-0.8	2.1	-0.7	2.2
4	-2.0	3.0	-1.8	3.4	-0.9	2.1	-0.8	2.4
5	-2.0	2.8	-1.7	3.0	-0.8	2.1	-0.7	2.4
6	-1.9	2.6	-1.5	3.0	-0.8	2.0	-0.8	2.4
7	-1.9	2.6	-1.6	3.0	-0.7	1.9	-0.6	2.2
8	-1.9	2.6	-1.6	3.0	-0.8	2.0	-0.7	2.4
9	-1.6	2.2	-1.1	3.0	-0.7	1.9	-0.6	2.1
10	-2.0	2.8	-1.8	4.0	-0.8	2.0	-0.7	2.4
11	-1.9	2.6	-1.4	2.6	-0.7	2.0	-0.7	2.2
12	-2.0	2.6	-1.6	3.0	-0.8	2.0	-0.6	2.2
13	-1.8	2.6	-1.6	3.0	-0.9	2.0	-0.8	2.4
14	-1.8	2.9	-1.6	3.0	-0.9	2.0	-0.7	2.2
15	-2.0	2.8	-1.8	2.8	-0.7	1.9	-0.6	2.2
16	-2.0	2.2	-1.7	2.0	-0.6	2.0	-0.5	2.4
17	-2.0	2.1	-1.6	2.0	-0.5	2.0	-0.4	2.6
18	-1.9	2.6	-1.4	3.2	-0.8	2.0	-0.7	2.2
19	-2.4	2.0	-2.1	2.0	-0.5	2.0	-0.4	2.8
20	-2.2	2.0	-1.4	2.4	-0.7	1.9	-0.5	2.1
21	-1.4	2.4	-1.2	3.0	-0.7	1.9	-0.5	2.1
22	-1.8	2.0	-1.9	2.0	-0.5	2.0	-0.4	2.0

Table S2: Parameters used to call the `C` executable implementation of DDA. This applies an SVD algorithm to obtain model features and the least squares error ($\{a_1, a_2, a_3, \rho\}$) for an input sequence x and model 2 in Supplementary Table S4

variable	value
`TAU_FILE`	`DELAY_FILE`
`DATA_FN`	`RandomWalk_Chr1.ascii`
`OUT_FN`	`DDA_OUT/Chr1_start_end_bin#.ascii`
`WL`	99942
`WS`	99942
`SELECT`	`ST CT 0 0`
`CT_CH_list`	$b_s\ b_{s+1}\ b_s\ b_{s+2} \dots b_s\ b_{N_{bins}}$

- `DELAY_FILE` ... file with 2 columns containing values of delay values τ_1, τ_2
- `RandomWalk_Chr1.ascii` ... file containing N_{bins} input sequences of length 100kbp ($N_{bins} = 2490$ for chromosome 1)
- `OUT_FN` ... file to store outputs of DDA
- `WL` ... window length from which $\{a_1, a_2, a_3, \rho\}$ are extracted from input data
- `WS` ... window shift
- `SELECT` ... for ST DDA: `1 0 0 0`; for CT DDA: `0 1 0 0`;
- $b_s\ b_{s+1}\ b_s\ b_{s+2} \dots b_s\ b_{N_{bins}}$ ... list of bins to CT

Table S3: Structure selection results for DNA-DDA. Performance is given for the delay pair τ_1, τ_2 that resulted in the highest correlation coefficient r_{PC} between $\text{PC}_{\text{DNA-DDA}}$ and $\text{PC}_{\text{Hi-C}}$ on chr22:16200000:36200001 (Figure S2).

Cell type	τ_1	τ_2	$r_{PC_{Chr22SS}}$	$r_{PC_{Chr22ALL}}$
GM12878	3	6	0.49	0.49
K562	11	50	0.42	0.32
hESC	2	4	0.21	0.53
IMR90	1	36	0.35	0.30

Table S4: All possible three term DDA models up to cubic nonlinearity and two delays. A checkmark "$\checkmark$" denotes term a_i in Eq. to be nonzero. Symmetric models are labeled with "S" and single delay models with "1". All others have two non-interchangable delays.

ModelNr	x_1	x_2	x_1^2	x_1x_2	x_2^2	x_1^3	$x_1^2x_2$	$x_1x_2^2$	x_2^3	model type
1	$\checkmark$	$\checkmark$	$\checkmark$	0	0	0	0	0	0	
2	$\checkmark$	$\checkmark$	0	$\checkmark$	0	0	0	0	0	S
3	$\checkmark$	$\checkmark$	0	0	0	$\checkmark$	0	0	0	
4	$\checkmark$	$\checkmark$	0	0	0	0	$\checkmark$	0	0	
5	$\checkmark$	0	$\checkmark$	$\checkmark$	0	0	0	0	0	
6	$\checkmark$	0	$\checkmark$	0	$\checkmark$	0	0	0	0	
7	$\checkmark$	0	$\checkmark$	0	0	$\checkmark$	0	0	0	1
8	$\checkmark$	0	$\checkmark$	0	0	0	$\checkmark$	0	0	
9	$\checkmark$	0	$\checkmark$	0	0	0	0	$\checkmark$	0	
10	$\checkmark$	0	$\checkmark$	0	0	0	0	0	$\checkmark$	
11	$\checkmark$	0	0	$\checkmark$	$\checkmark$	0	0	0	0	
12	$\checkmark$	0	0	$\checkmark$	0	$\checkmark$	0	0	0	
13	$\checkmark$	0	0	$\checkmark$	0	0	$\checkmark$	0	0	
14	$\checkmark$	0	0	$\checkmark$	0	0	0	$\checkmark$	0	
15	$\checkmark$	0	0	$\checkmark$	0	0	0	0	$\checkmark$	
16	$\checkmark$	0	0	0	$\checkmark$	$\checkmark$	0	0	0	
17	$\checkmark$	0	0	0	$\checkmark$	0	$\checkmark$	0	0	
18	$\checkmark$	0	0	0	$\checkmark$	0	0	$\checkmark$	0	
19	$\checkmark$	0	0	0	$\checkmark$	0	0	0	$\checkmark$	
20	$\checkmark$	0	0	0	0	$\checkmark$	$\checkmark$	0	0	
21	$\checkmark$	0	0	0	0	$\checkmark$	0	$\checkmark$	0	
22	$\checkmark$	0	0	0	0	$\checkmark$	0	0	$\checkmark$	
23	$\checkmark$	0	0	0	0	0	$\checkmark$	$\checkmark$	0	
24	$\checkmark$	0	0	0	0	0	$\checkmark$	0	$\checkmark$	
25	$\checkmark$	0	0	0	0	0	0	$\checkmark$	$\checkmark$	
26	0	0	$\checkmark$	$\checkmark$	$\checkmark$	0	0	0	0	S
27	0	0	$\checkmark$	$\checkmark$	0	$\checkmark$	0	0	0	
28	0	0	$\checkmark$	$\checkmark$	0	0	$\checkmark$	0	0	
29	0	0	$\checkmark$	$\checkmark$	0	0	0	$\checkmark$	0	
30	0	0	$\checkmark$	$\checkmark$	0	0	0	0	$\checkmark$	
31	0	0	$\checkmark$	0	$\checkmark$	$\checkmark$	0	0	0	
32	0	0	$\checkmark$	0	$\checkmark$	0	$\checkmark$	0	0	
33	0	0	$\checkmark$	0	0	$\checkmark$	$\checkmark$	0	0	
34	0	0	$\checkmark$	0	0	$\checkmark$	0	$\checkmark$	0	
35	0	0	$\checkmark$	0	0	$\checkmark$	0	0	$\checkmark$	
36	0	0	$\checkmark$	0	0	0	$\checkmark$	$\checkmark$	0	
37	0	0	$\checkmark$	0	0	0	$\checkmark$	0	$\checkmark$	
38	0	0	$\checkmark$	0	0	0	0	$\checkmark$	$\checkmark$	
39	0	0	0	$\checkmark$	0	$\checkmark$	$\checkmark$	0	0	
30	0	0	0	$\checkmark$	0	$\checkmark$	0	$\checkmark$	0	
41	0	0	0	$\checkmark$	0	$\checkmark$	0	0	$\checkmark$	S
42	0	0	0	$\checkmark$	0	0	$\checkmark$	$\checkmark$	0	S
43	0	0	0	0	0	$\checkmark$	$\checkmark$	$\checkmark$	0	
44	0	0	0	0	0	$\checkmark$	$\checkmark$	0	$\checkmark$	

Table S5: Omitted genomic bins. Includes centromeres from UCSD genome browser and low coverage bins of HiC contact maps in GM12878 (less than 10% of overall interaction probability).

chr1	1:5, 1219:1250, 1253:1431, 1442:1444, 1447:1448, 1463, 1488
chr2	895:895, 906:914, 921:941, 944, 1099, 1105
chr3	907:937
chr4	497:518
chr5	464:501, 698:699
chr6	585:602
chr7	581:617, 621:621
chr8	1, 440:459
chr9	393, 400, 433:605, 607, 609, 611, 623, 631:632, 635:635, 643, 647, 651:652, 659, 665:666, 680:682
chr10	396:417, 479
chr11	1, 510:545
chr12	347:372
chr13	1:181
chr14	1:190, 196
chr15	1:220
chr16	185, 332:333, 344, 363:463
chr17	1, 228:267
chr18	154:209
chr19	244:272
chr20	264:301
chr21	1:50, 55:60, 63:63, 67:68, 71:72, 75:77, 82:82, 84:84, 95:95, 108:130
chr22	1:105, 109, 112, 129:152

Table S6: Omitted genomic bins. Includes centromeres from UCSD genome browser and low coverage bins of HiC contact maps in K562 (less than 10% of overall interaction probability).

chr1	1:5, 1219:1250, 1253:1431, 1442:1444, 1447:1449, 1463, 1488
chr2	895, 906:914, 921:941, 944:945, 1099, 1105:1106
chr3	907:937
chr4	497:518
chr5	464:501, 698:699, 703, 707
chr6	1, 585:602,
chr7	581:617, 621
chr8	1, 440:459,
chr9	209:265, 287:316, 393, 400, 405, 407, 427, 433:605, 607:611, 615, 622:627, 631:632, 635, 643, 647:648, 651:652, 659, 665:666, 673, 676, 680:682
chr10	396:417, 1020
chr11	1, 510:545
chr12	347:372
chr13	1:181
chr14	1:183, 189, 196
chr15	1:198
chr16	185:185, 328, 332:333, 344:345, 363:463
chr17	1, 228:267
chr18	154:209
chr19	1, 244:272
chr20	264:301
chr21	1:50, 55:60, 63, 67:68, 71:72, 75:77, 81:84, 95:95, 108:130
chr22	1:106, 109, 129:152

Table S7: Omitted genomic bins. Includes centromeres from UCSD genome browser and low coverage bins of HiC contact maps in IMR90 (less than 10% of overall interaction probability).

chr1	1:7, 1203, 1207:1209, 1214, 1217:1250, 1253:1435, 1440:1444, 1447:1452, 1454, 1462:1464, 1486:1489, 1493:1496
chr2	870, 878, 895:896, 898, 905:914, 921:941, 944:945, 975, 1099:1101, 1104:1106, 1306
chr3	907:937
chr4	93, 495, 497:518
chr5	464:501, 697:710, 712:713
chr6	1, 585:602
chr7	581:617, 731:732, 749, 752:754, 1437
chr8	1:2, 73, 76:78, 80:81, 123, 440:459, 858
chr9	393:394, 397:398, 400:401, 403:407, 427, 433:605, 607:615, 619:628, 630:633, 635, 643:644, 646:648, 651:653, 658:668, 672:683
chr10	396:417, 420:421, 479, 481
chr11	1:2, 510:545, 900
chr12	347:372
chr13	1:181
chr14	1:190, 196
chr15	1:222, 224:225, 286:287, 305:306, 325, 843, 845
chr16	164, 184:185, 215, 288, 296, 328, 332:335, 344:345, 363:463
chr17	1, 192, 228:267, 364:364, 380:382, 464, 466
chr18	154:209
chr19	1:2, 244:272
chr20	264:301, 312
chr21	1:52, 55:63, 65:72, 75:79, 81:85, 87, 95, 103, 108:130, 138, 435, 442
chr22	1:106, 109, 112, 118, 123, 129:153, 164, 183:188, 213:214

Table S8: Omitted genomic bins. Includes centromeres from UCSD genome browser and low coverage bins of HiC contact maps in hESC (less than 10% of overall interaction probability).

chr1	1:8, 1203, 1207:1209, 1213:1214, 1217:1250, 1253:1431, 1434:1435, 1438:1452, 1454:1455, 1462:1464, 1486:1489, 1492:1496
chr2	870, 878, 894:896, 898, 905:914, 921:941, 944:945, 975, 1099:1101, 1104:1106, 1306
chr3	907:937, 1629
chr4	495, 497:518
chr5	464:501, 697:710, 712:713
chr6	1, 585:602
chr7	581:617, 621, 731:733, 749:750, 752:754, 1026, 1437
chr8	1:2, 73, 76:78, 81, 440:459, 858
chr9	393, 397:407, 412, 415:417, 419, 427, 433:605, 607:616, 618:628, 630:636, 643:644, 646:649, 651:656, 658:667, 672:683
chr10	396:417, 479
chr11	1:2, 510:545
chr12	347:372
chr13	1:181, 1118
chr14	1:186, 188:190, 194:196
chr15	1:198, 201, 206, 208:209, 211:212, 214:222, 224:225, 233, 305:306, 325, 843, 845
chr16	148, 164, 184, 215, 288, 296, 303, 328, 331:335, 344:345, 363:463
chr17	1, 192:192, 228:267, 364, 380:382, 464
chr18	154:209
chr19	1:2, 244:272
chr20	264:301, 312
chr21	1:52, 55:63, 65:72, 75:79, 81:84, 86:87, 95, 103, 108:130, 138, 435:435, 442:442
chr22	1:107, 109, 112, 129:156, 158:159, 183:188, 214

Table S9: Principal components used to determine A/B compartments from Hi-C and DNA-DDA matrix respectively

Chr	GM12878		K562		IMR90		hESC	
	Hi-C	DNA-DDA	Hi-C	DDA	Hi-C	DDA	Hi-C	DDA
1	1	1	1	1	2	1	1	4
2	1	1	1	1	2	1	1	1
3	1	1	2	1	2	1	3	1
4	1	1	2	1	2	1	4	1
5	1	1	2	1	2	1	3	1
6	1	1	1	1	2	2	4	1
7	1	1	1	1	2	1	2	1
8	1	1	1	1	2	1	4	1
9	1	2	2	1	2	2	2	1
10	1	1	1	1	2	2	3	1
11	1	2	1	1	2	2	2	1
12	1	1	1	1	1	2	2	1
13	1	1	1	1	1	1	4	1
14	1	2	1	1	1	2	3	1
15	1	1	1	1	1	1	2	1
16	1	2	1	1	1	2	4	1
17	1	2	1	1	1	2	2	1
18	1	1	1	1	1	2	4	1
19	1	2	1	1	1	1	1	2
20	1	2	1	1	1	2	2	1
21	1	2	1	2	1	2	2	2
22	1	2	1	2	1	2	2	4

Table S10: Overall datasets used by comparison methods

SACSANN	GSE35156	mESC,hESC
	GSE96107	mESC,NPC,CN
	GSE59027	mESC,NPC,CN
ABCNet	GSE35156	hESC
	GSE48592,GSE63525	EBV
	GSE43070,GSE63525	IMR90
	GSE31388	Dnase EBV
	GSE31263	Dnase IMR90
	GSE53261	Hi-C meth fibroblast
	http://www.roadmapepigenomics.org/	Hi-C meth EBV
Orca	4DNFI9GMP2J8	H1ESC
	4DNFI643OYP9	HFF
	4DNFILP99QJS	cohesin-depleted HCT116
DNA-DDA	GSE63525	GM12878,K562
	GSE35156	hESC,IMR90

Table S11: Regions used for Orca comparison.

1	chr9:5504001-37504000
2	chr9:69888001-101888000
3	chr9:104960001-136960000
